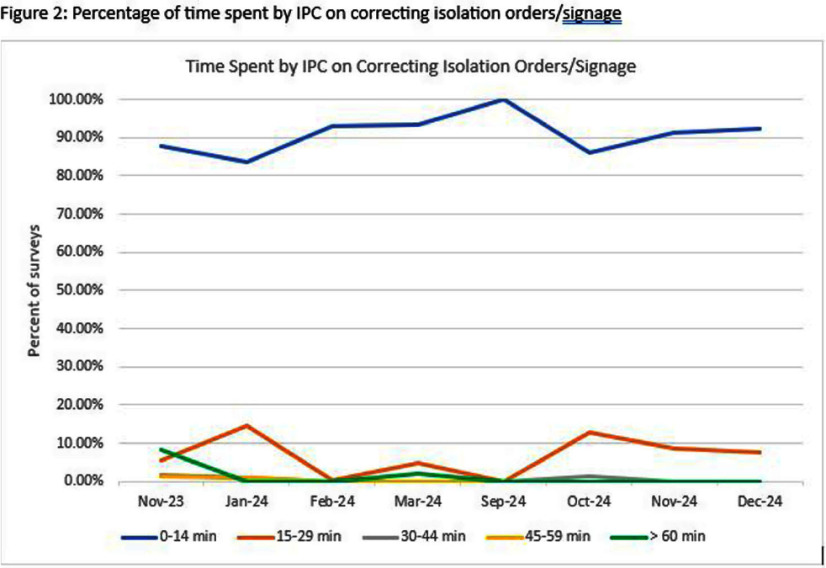# Impact of Automated Isolation Precautions Orders on Infection Preventionists’ Surveillance and Rounding Time at a Pediatric Hospital

**DOI:** 10.1017/ash.2025.397

**Published:** 2025-09-24

**Authors:** Christelle Ilboudo, Elizabeth Monsees, La Sonya Buford

**Affiliations:** 1 Children’s Mercy Kansas City; 2 Children’s Mercy Hospital; 3 Children’s Mercy Hospital Kansas City

## Abstract

**Background:** Seasonal viral respiratory pathogens present a significant clinical burden to pediatric patients. During the viral season, hospitals face an increased number of patients requiring isolation precautions. In surveying isolation practices among pediatric institutions, we identified a high variation in interpreting and implementing isolation precautions, including the duration of isolation. This variability resulted in practice challenges articulated by Infection Prevention and Control (IPC) and clinical teams. We sought to simplify and reduce wasteful work processes. Through the initial phase of this quality improvement project, we examined the congruency between isolation orders and signage and the IPC surveillance time needed to modify isolation practices. **Method:** Our interdisciplinary team developed and created a process map of isolation work processes, identifying at least 7 decision points to place isolation or to de-isolate hospitalized patients. A prioritization matrix was used to select drivers for maximum impact: 1) initiate empiric isolation for the most common viral respiratory pathogens and 2) develop and implement a tool for de-isolation. Improvement measures included isolation order and signage appropriateness (outcome), modifications of isolation orders by providers (process), and IPC time for correcting isolation or providing just-in-time training during rounds (balancing). Both outcome and balancing measures were captured using an investigator-developed survey, which was streamlined 3 times. Comprised of 25 questions, the survey is completed throughout the month by IPC during surveillance and environmental rounds, with collection spanning 2 viral seasons. Descriptive statistics are used to analyze the data for trending and practice modifications. **Result:** We completed 929 individual observations via survey over 8 months. The appropriateness of isolation precautions orders improved over time, with a shift in the center line (Figure 1). We identified that the rate of appropriateness decreased at the height of the viral respiratory season due to additional precautions (droplet isolation for certain viruses based on the risk of splashes or sprays per our policies). Surveillance time for correcting precaution signs and/or orders decreased from ≥ 15 to ≤ 5 minutes (Figure 2). **Conclusion:** Automation and use of empiric isolation precautions orders for the most common viral respiratory pathogens in our hospitalized patients has led to a reduction in wasteful workflow processes, minimized decision points, and has decreased IPC time spent correcting isolation orders and signs. In the project’s next phase, we hope to minimize patients’ time in isolation by using a nurse-driven tool to assess their clinical readiness for de-escalation of isolation during their hospitalization.

**Figure:**
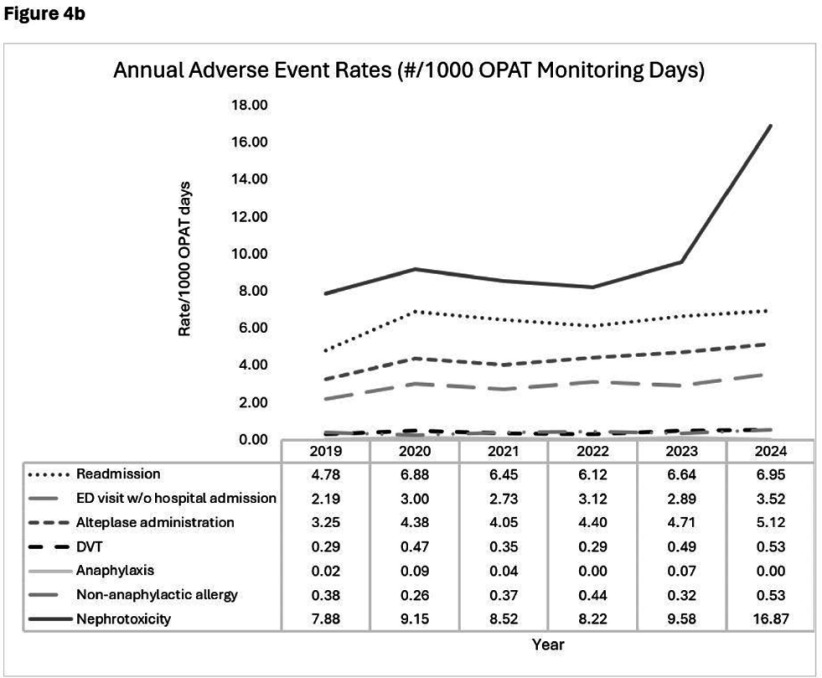

**Figure:**